# Declared Intention to Vaccinate against COVID-19 and Actual Vaccination—The Role of Trust in Science, Conspiratorial Thinking and Religiosity

**DOI:** 10.3390/vaccines11020262

**Published:** 2023-01-25

**Authors:** Józef Maciuszek, Mateusz Polak, Katarzyna Stasiuk, Jerzy Rosiński

**Affiliations:** 1 Institute of Applied Psychology, Faculty of Management and Communication, Jagiellonian University, 31-007 Krakow, Poland; 2 Institute of Economy, Finance and Management, Faculty of Management and Communication, Jagiellonian University, 31-007 Krakow, Poland

**Keywords:** COVID-19 vaccination, willingness to vaccinate, actual vaccination, trust in science, conspiratorial thinking, religiosity

## Abstract

AIMS: The study aims to investigate how trust in science, conspiratorial thinking, and religiosity affected people’s declared willingness to vaccinate against COVID-19 at the onset of the vaccination program in Poland, their actual vaccination, and the consistency between intention and vaccination. METHODS: In a longitudinal design, a representative sample of 918 members of the Polish general population was polled at the beginning of the vaccination program (February 2021) and polled again after 6 months of mass vaccination (August 2021). We measured the willingness to vaccinate, actual vaccination after 6 months, and individual variables—trust in science, conspiratorial thinking and religiosity. RESULTS: The actual vaccination rate was higher than the declared intent, especially in the initially undecided and unwilling groups. Higher Trust in science and lower Conspiratorial Thinking were associated with declared intent to vaccinate and actual vaccination, while Religiosity was not clearly associated with vaccination. CONCLUSIONS: Declared willingness to vaccinate is not an effective indicator of actual vaccination. Trust in science and Conspiratorial thinking are important factors associated with vaccine hesitancy. There may be a possibility to influence those unwilling to vaccinate and that are undecided to eventually get vaccinated.

## 1. Introduction

In the last decade before the outbreak of the COVID-19 pandemic, there was growing skepticism about vaccinations in many countries, including Poland, manifested by a rise in the activity of anti-vaccine movements and a decline in vaccination rates [1,2]. In December 2020, it was announced in Poland that mass vaccination against COVID-19 would begin in February 2021. This raised hopes for a rapid containment of the pandemic, but also doubts about the willingness of the vast majority of the population to undergo vaccination. These concerns were supported by the results of surveys on the intention to vaccinate against COVID-19. In a survey conducted in November 2020, only 36% of respondents said they would choose to vaccinate, while 47% said they would not, and 17% were undecided [3].

This low rate of declared readiness for COVID-19 vaccination in the months prior to the start of the mass vaccination program [3] prompted us to design a longitudinal study to examine the relationship between the intention expressed by respondents one week before the start of the COVID-19 vaccination program (Willing to vaccinate vs. Unwilling vs. Undecided) and their actual behavior (getting vaccinated or not). The second measurement was carried out after 6 months, when the entire population had an opportunity to get vaccinated.

Research on anti-vaccine attitudes shows that skepticism toward vaccination, beliefs, emotions and behaviors regarding vaccines and vaccination are influenced by many factors [4,5,6]. Among them, researchers list science rejection, a tendency toward conspiracy thinking, and religiosity [4]. However, many studies have examined the impact of these factors only on attitudes toward vaccination or declarations of vaccination, not actual behavior. In contrast, we conducted a study in which we examined how these factors (attitudes toward science, generic conspiratorial thinking, and religiosity) affect not only the declared intention to vaccinate or not vaccinate, but also actual behavior, and the consistency between intention and behavior. We were also interested in whether any inconsistency between the declaration and subsequent behavior would correlate with changes in attitudes toward science and in the level of conspiracy thinking. We wanted to see how the relationship between declaration and behavior would be related to select variables which have traditionally been considered predictors of vaccine rejection.

### 1.1. Science Rejection, Religiosity, Conspiracy Beliefs and the COVID-19 Pandemic

#### 1.1.1. Science Rejection and COVID-19 Pandemic

Anti-science is the deliberate rejection of established scientific knowledge without any basis or scientific evidence [6,7]. Anti-scientific attitudes include science rejection, manifested by a lack of trust in science in general or questioning its specific findings (e.g., vaccine rejection, climate change denial, intuitive opposition to GMOs), and pseudoscience—the proclamation and support of theories that contradict modern scientific knowledge or are not supported by scientific research (e.g., creationism, homeopathy). These phenomena accompany the current pandemic—after its outbreak we were confronted with many false beliefs about the nature of the SARS-CoV-2 virus, treatment options, etc. Following Lewis [8], let us list some examples of myths related to the pandemic and vaccination which were present during the early onset of COVID-19: (a) COVID-19 is no worse than the flu; (b) We don’t need to wear a mask; (c) Hydroxychloroquine is an effective treatment; (d) Spikes in cases are because of increased testing; (e) Any vaccine will be unsafe and a bigger risk than getting COVID-19; and (f) We can achieve herd immunity by letting the virus spread through the population. Such anti-scientific attitudes and false beliefs are mainly related to ideology and worldview (deep religiosity, conspiracy beliefs, political orientation) and morality (moral concerns about what scientists do and about their discoveries) [5,9].

#### 1.1.2. On Religiosity in the Context of the Dispute with Science and the Current Pandemic

Religion and science have been in natural opposition for centuries (e.g., the rejection of the heliocentric theory and of Darwin’s theory of evolution had religious premises). Both science and religious faith offer very complex and comprehensive systems of understanding the world, which compete not only in the sphere of explaining the meaning of life, but also in the sphere of professed values and morality [10,11]. It has been proven that religiosity is associated with lower levels of scientific knowledge, with more negative attitudes toward science and less trust in scientific sources of information [11]. From the point of view of our project, it is important to emphasize that a number of studies conducted before the outbreak of the current pandemic indicated that religiosity positively correlates with skepticism toward vaccination. This correlation is most likely more due to the generally lower trust in science among religious believers than due to moral issues [9].

Research on the relationship between religion and socio-psychological functioning during the COVID-19 pandemic provided interesting results. First, the assumption that crises and difficult situations tend to increase religiosity was confirmed. Second, religiosity may positively correlate with resistance to preventative measures, including vaccination during the current pandemic. In the U.S., 28% of respondents confirmed an increase in their sense of religiosity due to the pandemic [12], and a survey of 1000 Polish adults conducted at the start of the pandemic found that a large percentage of respondents spend more time than before on prayer and other religious practices [13]. The same study indicated that devoting more time to religious practices was associated with disregarding certain government restrictions, having less knowledge about COVID-19, and being more inclined to believe in conspiracy theories. As has been found in other studies [14], only the most dogmatic and fundamentalist type of religiosity can lead to conspiracy beliefs about the coronavirus.

#### 1.1.3. Coronavirus Conspiracy Beliefs

Before the outbreak of the COVID-19 pandemic, there existed widespread conspiracy theories and false information about the side effects of vaccination in general. One of the most widespread conspiracy theories about vaccination is that the real information about its effects and consequences is not available to the public, and the harmfulness of vaccines is hidden from the people, allowing pharmaceutical companies to get rich [15]. Among the most high-profile false messages is the claim that autism is a side effect of vaccination [16].

Earnshaw and his team [17] showed that people are most prone to succumb to conspiracy theories when they feel anxious, unable to influence the events around them. This tendency mostly occurs in times of crisis and during events which have a significant impact on people’s lives (such as pandemics). This was confirmed by a study by Šrol and colleagues [18], which found that people who feel more anxious and out of control during the current pandemic are more likely to believe various conspiracy theories about COVID-19, as well as pseudoscientific theories unrelated to the pandemic. Conspiracy thinking is also influenced by a lack of trust in the institutions tasked with fighting the pandemic and in scientific findings about it. A study by Kim and Kim [19] found that authoritarianism, religiosity, trust in social networks, perceived risk, fear, negative emotions, and attribution of blame positively influence beliefs in conspiracy theories.

At the beginning of the pandemic, the following misinformation and conspiracy theories were most prevalent among US citizens: (a) the number of coronavirus-related deaths was exaggerated; (b) information about the COVID-19 threat is being manipulated by the government; (c) the coronavirus was deliberately created so that certain people could rule the world; and (d) the disease is being used to create a dangerous vaccine. It has also been fairly widely assumed that the pandemic was caused by Bill Gates for personal gain [20]. These theories are particularly dangerous because they directly affect adherence to medical and government recommendations aimed at reducing the spread of the virus. The studies found that people influenced by conspiracy theories knew less about the new vaccine, were more skeptical of medical professionals, were less likely to act on recommendations to reduce the spread of the virus, and were reluctant to be vaccinated against COVID-19 [17]. Belief in conspiracy theories about the pandemic was associated with a decreased likelihood of getting tested for the coronavirus, and a lower tendency to adhere to recommendations even in case of an increased threat of exposure to the virus [21,22], which could lead to health risks for the entire community.

The above results relate to the impact of specific COVID-19 conspiracy beliefs on attitudes toward the COVID-19 pandemic and vaccination. Moskovici [23] distinguished the belief in specific conspiracy theories from a general tendency toward conspiratorial thinking (the conspiracy mentality). Research indicates that people who believe in one particular conspiracy theory tend to believe in others more, even when they involve unrelated events [7,24]. Therefore, it is reasonable to expect that general conspiratorial thinking would be associated with rejection of the COVID-19 vaccine as well.

### 1.2. Current Research

The current research aims to answer the following questions:

The primary question was whether there exists a relationship between the selected individual variables (science rejection, religiosity, conspiracy beliefs), the declared willingness to vaccinate against COVID-19 (willing, unwilling, undecided), and actual COVID-19 vaccination (vaccinated vs. unvaccinated vs. undecided). Additionally, we wanted to investigate the relation between these individual variables and the congruence between intended and actual vaccination.

Moreover, we investigated whether there would be changes in attitudes toward science and conspiracy thinking between the first and second measurements; what the dynamics of these changes would be; and whether changes between declaration (intention) and behavior during the implementation of the vaccination program go hand in hand with a change in attitudes toward science and conspiratorial thinking.

The presented results also investigate the relationship between the declared willingness to be vaccinated against COVID-19 before the start of the vaccination program (yes vs. no vs. undecided) and the actual behavior during the vaccination program (vaccinated vs. not vaccinated). This is necessary for the above research questions, however this result has already been published in a previous paper in *Vaccines* [25], so it does not warrant a separate research question here.

## 2. Materials and Methods

### 2.1. Procedure and Participants

A representative sample of the Polish general population took part in the longitudinal study with two measurements. A random quota sampling method was used based on sex (two subgroups), age (five subgroups) and place of residence (five subgroups); each demographic criterion was controlled to be representative of the Polish general population, giving a total of 50 weighted cells. Weights were calculated based on these three demographic criteria, and participants were drawn randomly from cells to fit demographic quotas. The study was conducted online by the Ariadna Nationwide Research Panel, a company specializing in the polling of large samples for the purposes of research. The panel allows random sampling from among 300,000 registered and verified people. The socio-demographic profile of those registered in the panel corresponds to that of Polish Internet users.

In a longitudinal design, the first measurement was conducted in February 2021 (Time 1), and we reached the same group of respondents in August 2021 (Time 2). Reaching the same sample for a second time was possible, as Ariadna uses unique identifiers for their respondents and there is a possibility to invite particular respondents to a survey.

For participating in the survey, respondents received credit points that could be exchanged for gifts.

### 2.2. Instruments

Questionnaires in two measurements were presented online on the same survey platform. The questionnaire consisted of single-choice items which required on average 10 min to be completed, and it contained the following parts:

(a) Demographic data, including age, gender, education, and place of residence;
(b) Trust in science, which was measured with nine statements (e.g. “Science allows us to understand the Universe better than religion”, “We can only rationally believe what can be proven scientifically”, “Science tells us everything about what makes up reality”). These statements were based on Farias and Reiman’s Belief in Science scale [10]. The responses to statements were measured on a 5-point Likert scale from 1 (definitely do not agree) to 5 (definitely agree). The Cronbach’s alpha at Time 1 and Time 2 was the same at α = 0.921;
(c) Conspiratorial thinking, measured using 10 statements taken from the Generic Conspiracy Beliefs Scale [24,26]. The Generic Conspiracy Beliefs Scale aims to study generalized tendencies to believe in conspiracy theories without reference to a specific theory, including such items as “Above the leaders of states, there are a small number of clandestine groups that maintain real control over world politics”, “Certain viruses or diseases are spread as a result of deliberate, covert action by certain organizations”, or “Groups of scientists manipulate, prepare or hide facts to deceive the public”). We removed questions pertaining to the Government malfeasance and Extraterrestrial beings subscales, since they are too extreme and are the least relevant to vaccine-related beliefs. The responses were measured on a 5-point Likert scale from 1 (definitely not true) to 5 (definitely true). The Cronbach’s alpha in Time 1 and in Time 2 was 0.929;
(d) Religiosity, which was measured by 10 statements (e.g. “Salvation is the goal of my life”, “I participate in the cultural life organized by the Church”, “I am striving to receive the grace of God”; religiosity scale by Socha [27]). The responses were measured on a 5-point Likert scale from 1 (definitely do not agree) to 5 (definitely agree), allowing the answer “not applicable”, which was subsequently treated as missing data. Religiosity was measured only at Time 1, as theory shows it to be stable over time, while Trust in science and Conspiratorial thinking were measured at Time 1 and Time 2, as we assumed that both of these factors may change during the pandemic. The Cronbach’s Alpha for religiosity was 0.944;
(e) At Time 1 we also measured the declared willingness to be vaccinated ("Will you get vaccinated for coronavirus?") with responses of “no”, “yes”, and “undecided”. At Time 2, we asked if respondents actually got vaccinated, with responses of “no”, “yes”, and “still undecided”.

### 2.3. Ethical Considerations

The study protocol was reviewed and approved by the Ethics Committee of the Institute of Applied Psychology, Jagiellonian University in Krakow (Poland). The questionnaire collected no identifying personal data from the participants.

### 2.4. Data Analysis

IBM SPSS Statistics (SPSS) software version 26 was used to perform the statistical tests: repeated measures ANOVA and multiple linear regression.

## 3. Results

### 3.1. Drop-Out of Participants

At Time 2, some of the original 1391 participants dropped out, therefore the final sample was N = 918, meaning a 34% dropout rate due to random reasons. There were 558 women and 360 men in the final sample. A total of 36% of them were aged 18–34, 41% were aged between 35–54, and 23% were aged 55 or older. Moreover, 10% had a primary or vocational education, 42% had a secondary or postsecondary education, and 38% had a Bachelor’s or Master’s degree.

### 3.2. Consistency and Shift between Declared and Actual Vaccination

We compared the declared intention to (not) vaccinate against COVID-19 at Time 1 with the actual action taken at Time 2. At Time 1, the sample included 225 participants unwilling to vaccinate, 404 willing to vaccinate and 289 undecided. At Time 2, there were 162 unvaccinated, 567 vaccinated and 137 undecided participants (43 subjects were excluded because they provided ambiguous answers, such as *Not yet but I am going to get vaccinated*, *I took one dose but don’t know if I will take the second one* and *I took one dose but I don’t want to take the second one*). A McNemar-Bowker test indicated a significant shift (χ^2^ (3, N = 875) = 161.612, *p* < 0.001, Cramer’s V = 0.519) toward vaccination between declaration and behavior (This particular result has already been published in Maciuszek, Polak, & Stasiuk [25].): at Time 1, 24.5% of the sample were unwilling to vaccinate vs. 18.5% unvaccinated at Time 2; the 31.4% undecided at Time 1 dropped to 15.6% at Time 2; and while 44% wanted to vaccinate at Time 1, this number grew to 64.8% actually vaccinated at Time 2.

### 3.3. Individual Differences and the Intention to Vaccinate

As a preliminary analysis, we ran correlation analyses between Conspiratorial thinking, Trust in science and Religiosity at Time 1. Higher Conspiratorial thinking was associated with higher Religiosity (r = 0.192, *p* < 0.001), higher Trust in science was associated with lower Religiosity (r = −0.313, *p* < 0.001) and higher Trust in science was weakly associated with lower Conspiratorial thinking (r = −0.066, *p* = 0.047).

We also ran correlation analyses between Conspiratorial thinking (Time 2), Trust in science (Time 2), and Religiosity (Time 1). Higher Conspiratorial thinking was associated with higher Religiosity (r = 0.210, *p* < 0.001), higher Trust in science was associated with lower Religiosity (r = −0.354, *p* < 0.001), and higher Trust in science was associated with lower Conspiratorial thinking (r = −0.123, *p* < 0.001).

We then ran similar correlation analyses for Time 1 in each individual group (willing-to-vaccinate, unwilling-to-vaccinate and undecided) and found that in the unwilling-to-vaccinate group, higher Religiosity was associated with lower Trust in science (r = −0.350, *p* < 0.001), but the correlation between Conspiratorial thinking and Religiosity disappeared (*p* = 0.699). In the Undecided group, higher Conspiratorial thinking was associated with higher Trust in science (sic!) (r = 0.176, *p* = 0.003), and higher Religiosity was associated with lower Trust in science (r = −0.174, *p* = 0.005) but there was no significant correlation between Conspiratorial thinking and Religiosity (*p* = 0.372). In the willing-to-vaccinate group, higher Conspiratorial thinking was associated with higher Religiosity (r = 0.281, *p* < 0.001) and higher Religiosity was associated with lower Trust in Science (r = −0.337, *p* < 0.001), but there was no significant correlation between Trust in science and Conspiratorial thinking (*p* = 0.069).

In order to investigate the differences in the measured individual variables at Time 1 (Trust in science, Conspiratorial thinking and Religiosity) between those willing/undecided/unwilling to vaccinate against COVID-19 at Time 1, an ANOVA was conducted. Results indicated significant differences between groups in Conspiratorial thinking (F(2,915) = 78.517; *p* < 0.001, η^2^ = 0.146), Trust in science (F(2,915) = 41.268, *p* < 0.001, η^2^ = 0.083) and Religiosity (F(2,821) = 12.173, *p* < 0.001, η^2^ = 0.029).

Pairwise comparisons (Bonferroni adjusted) indicated that there were significant differences (all *p* < 0.001) in Conspiratorial Thinking—it was the lowest in those willing-to-vaccinate (M = 3.00, SD = 0.80), highest in those unwilling-to-vaccinate (M = 3.77, SD = 0.05), and moderate in the undecided group (M = 3.44, SD = 0.67).

Moreover, significant differences were found in Trust in science—lower in those unwilling-to-vaccinate (M = 3.49, SD = 0.82) than those willing-to-vaccinate (M = 3.97, SD = 0.64; *p* < 0.001), and lower in the undecided (M = 3.61, SD = 0.68) than the willing-to-vaccinate group (*p* < 0.001). The difference between unwilling-to-vaccinate and undecided groups was not significant (*p* = 0.14).

**Figure 1:**
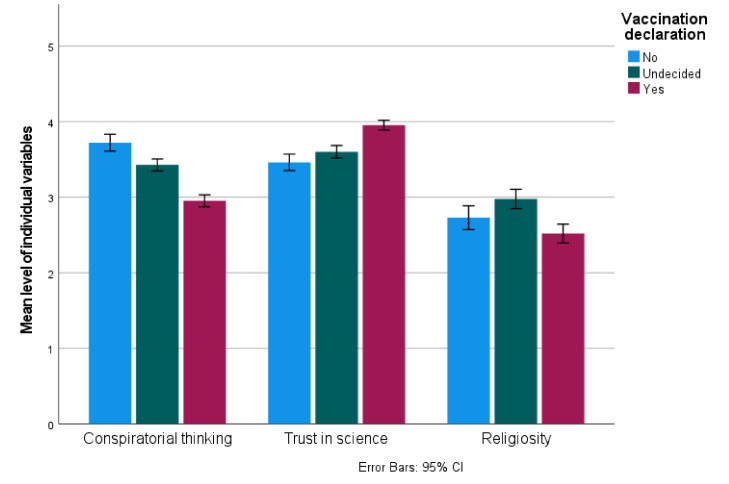
Individual differences and declared intention to vaccinate against COVID-19.

Religiosity was higher in the undecided (M = 2.98, SD = 1.04) than the willing-to-vaccinate group (M = 2.51, SD = 1.22; *p* < 0.001). There were no significant differences between the unwilling-to-vaccinate and undecided (*p* = 0.068) groups nor between the unwilling-to-vaccinate and willing-to-vaccinate (*p* = 0.112) ones. The results are presented in Figure 1.

### 3.4. Individual Differences and Actual Vaccination

We ran correlation analyses between Conspiratorial thinking (Time 2), Trust in science (Time 2), and Religiosity (Time 1) in each behavior group separately (vaccinated, unvaccinated and undecided). We found that in the Unvaccinated group, higher Religiosity was associated with lower Trust in science (r = −0.431, *p* < 0.001), while other correlations were nonsignificant. In the Undecided group, higher Trust in science was associated with higher Conspiratorial thinking (!) (r = 0.187, *p* = 0.029), while other correlations were nonsignificant. In the Vaccinated group, all correlations between the three variables were significant at *p* < 0.001: Religiosity × Conspiratorial thinking positive, r = 0.260, Religiosity × Trust in science negative, r = −0.370, Trust in science × Conspiratorial thinking negative r = −0.163.

We then investigated the differences between the measured individual variables (Trust in science T2, Conspiratorial thinking T2 and Religiosity T1) between those vaccinated, unvaccinated and undecided at Time 2. The measure of Religiosity was taken at Time 1 only, as this individual variable has been shown to be stable over time in longitudinal studies, even as religious attendance changes [28,29]. Conspiratorial thinking was highly correlated between Time 1 and Time 2 (r = 0.722, *p* < 0.001), as was Trust in science (r = 0.705, *p* < 0.001), but these cannot be considered stable over time.

An ANOVA was conducted, indicating significant differences between groups for Religiosity (F(2,781) = 3.761, *p* = 0.024, η^2^ = 0.01), Conspiratorial thinking (F(2,872) = 39.927, *p* < 0.001, η^2^ = 0.083) and Trust in science (F(2,872) = 29.182, *p* < 0.001, η^2^ = 0.063). Pairwise comparisons (Bonferroni adjusted) indicated that there were significant differences (all *p* < 0.02) in Conspiratorial thinking between the unvaccinated (M = 3.736, SD = 0.70), undecided (M = 3.480, SD = 0.67) and vaccinated (M = 3.133, SD = 0.86) groups—the highest level of Conspiratorial thinking was present in the unvaccinated group, and the lowest in the vaccinated group.

Trust in science was significantly lower in the unvaccinated (M = 3.453, SD = 0.88) than the vaccinated (M = 3.898, SD = 0.69) group (*p* < 0.001), and lower in the undecided (M = 3.574, SD = 0.68) than the vaccinated group (*p* < 0.001). The difference between unvaccinated and undecided was not significant (*p* = 0.454).

**Figure 2:**
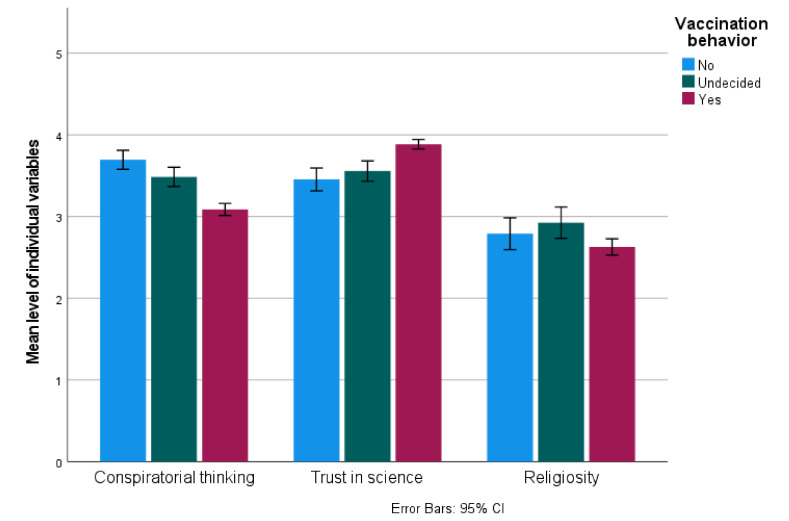
Individual differences and actual vaccination against COVID-19.

Religiosity was higher in the undecided (M = 2.92, SD = 1.07) than the vaccinated group (M = 2.63, SD = 1.17, *p* = 0.032), other comparisons were nonsignificant. The results are presented in Figure 2.

### 3.5. Individual Differences and the Consistency between Declaration and Behavior

We divided the sample based on both the declared intent to vaccinate at Time 1 and the actual behavior at Time 2, which resulted in nine possibilities: Consistently Pro-vaccine (YES/YES, N = 379; 43.3% of the sample), Consistently Anti-vaccine (NO/NO, N = 126; 14.4%), Consistently Undecided (UND/UND, N = 86; 9.8%); Pro-vaccine turned Anti-vaccine (YES/NO, N = 6; <1%), Pro-vaccine turned Undecided (YES/UND, N = 8; <1%); Anti-vaccine turned Pro-vaccine (NO/YES, N = 48; 5.4%), Anti-vaccine turned Undecided (NO/UND, N = 43; 4.9%); Undecided turned Pro-vaccine (UND/YES, N = 149; 17%), and Undecided turned Anti-vaccine (UND/NO, N = 30; 3,4%). Due to the low numbers, we dropped the YES/NO and YES/UND groups from further analyses.

An ANOVA of the remaining seven groups indicated significant differences in Religiosity (Time 1; F(6,764) = 5.790, *p* < 0.001, η^2^ = 0.034), Conspiratorial thinking (Time 2; F(6,854) = 14.995, *p* < 0.001, η^2^ = 0.147) and Trust in science (Time 2; F(6,854) = 8.633, *p* < 0.001, η^2^ = 0.106).

Pairwise comparisons (Bonferroni adjusted) indicated the lowest level of Conspiratorial thinking in the YES/YES group compared to all other groups (highest *p* = 0.022) and a higher level in the NO/NO than the UND/YES group (*p* = 0.004).

For Trust in science, it was the highest in the YES/YES group compared to all other groups (highest *p* = 0.005), with all other comparisons being nonsignificant.

**Figure 3:**
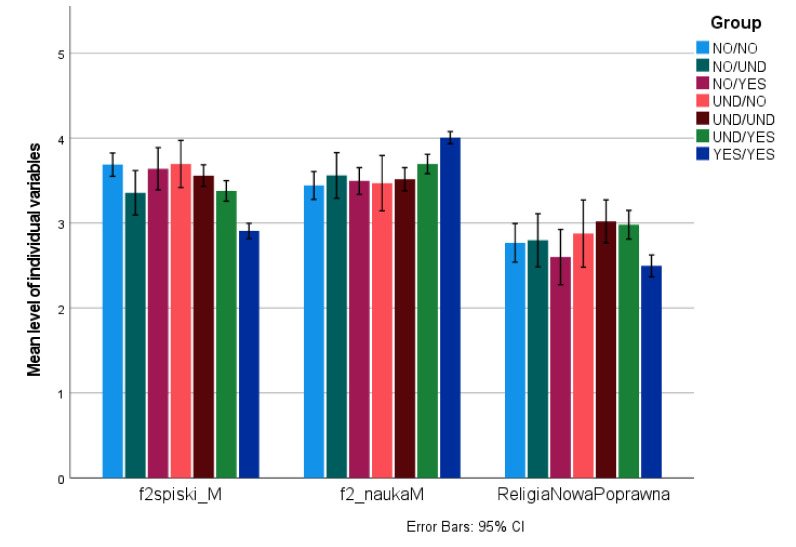
Individual differences and consistency between declared and actual vaccination against COVID-19.

For Religiosity, it was lower in the YES/YES group than the UND/YES (*p* < 0.001) and the UND/UND group (*p* = 0.006); all other comparisons were nonsignificant. Descriptive statistics are presented in Table 1, and the results are presented in Figure 3.

We also investigated whether consistency or inconsistency between declaration and behavior was associated with changes in individual differences (Conspiratorial thinking and Trust in science) between Time 1 and Time 2. Paired samples *t*-tests for individual groups indicated that Conspiratorial thinking was higher at Time 1 than at Time 2 in the NO/NO group (M = 3.87, SD = 0.75 vs. M = 3.75, SD = 0.72, t(125) = 2.408, *p* = 0.017), and Trust in science was higher at Time 1 in the UND/NO group (M = 3.70, SD = 0.76 vs. M = 3.53, SD = 0.80, t(29) = 2.430, *p* = 0.022), but higher at Time 2 in the YES/YES group (M = 3.98, SD = 0.65 vs. M = 4.03, SD = 0.67, t(378) = 1.990, *p* = 0.047).

## 4. Discussion

### 4.1. The Relationship between the Declaration before the Vaccination Program and the Actual Behavior during the Vaccination Program

To summarize the main results of the present research, we can divide them into three main parts: (1) intention to vaccinate, (2) actual vaccination behavior, and (3) the correspondence between intention and behavior.

As to the intention to vaccinate, the most consistent were those who declared that they would be vaccinated; 96.5% of them followed the expressed intention. Only six people (1.5 percent) acted contrary to the declaration and rejected vaccination and eight (2 percent) turned out to be undecided. This result is consistent with the results of other studies showing the congruence between intended and actual vaccination against COVID-19 by pro-vaccine individuals (e.g. [30]). In the initially unwilling-to-vaccinate group, 58% remained consistent, 22% got vaccinated and 20% of people became undecided. This result is inconsistent with the aforementioned study of Wang et al. [30], where more than half of people who declared their unwillingness to vaccinate against COVID-19 eventually got vaccinated. In our study, the largest change occurred in the group of people who were undecided at Time 1: 56% of them got vaccinated, and 11% chose to not get vaccinated, with 33% remaining undecided. Therefore, the results show an increase in pro-vaccine attitudes between declaration and behavior. First, a high percentage of undecided individuals eventually got vaccinated. Second, the consistency between declaration and behavior was significantly higher in pro-vaccine individuals. This may indicate a partial success of vaccine uptake promotion programs, which were able to persuade the undecided individuals, however they failed to persuade the already anti-vaccine group.

### 4.2. Relationship between Selected Individual Variables and Type of Intentions, as well as Actual COVID-19 Vaccination Behavior

In our research project, we were interested in what role Generic conspiratorial thinking, Negative attitudes toward science and Religiosity would play in shaping the relationship between expressed intention and actual behavior. We tested whether there is a positive relationship between these variables in the three groups distinguished by type of intention regarding upcoming COVID-19 vaccination (YES, NO, I DON’T KNOW). In these groups, there was a slightly different pattern of association between the individual variables studied. The results confirmed the negative relationship between trust in science and religiosity in each group. Regardless of the type of declaration regarding vaccination behavior, higher religiosity goes hand in hand with significantly lower trust in science. Six months after the start of COVID-19 vaccination, the same relationship occurred in both those who were vaccinated and those who rejected the vaccination. In contrast, the aforementioned relationship (the higher the religiosity, the lower the trust in science) did not occur in those who were undecided as to whether to receive the vaccine or not.

In the undecided groups, there was another different result regarding the correlation between variables. In undecided people, trust in science positively correlates with conspiracy thinking. It seems that this specific and atypical co-occurrence of belief in science and conspiracy thinking is a factor shaping people’s indecision both during the expression of intentions (when vaccination has not yet begun) and at the stage of implementation of the vaccination program (let us emphasize that the personal composition of these two groups was not identical: from the undecided group (Time 1),—32.5% remained undecided, and from the group of people expressing the intention not to vaccinate, 19.8% of people became undecided).

Many social behavior researchers make the assumption (e.g., Festinger, creator of the theory of cognitive dissonance [31]) that people strive for consistency between different beliefs about the same issue and for consistency between their views and behavior. Cognitive dissonance is a state of tension that arises from the discovery of discrepancies between one’s own beliefs or between beliefs and behavior. Such a state triggers the motivation to reduce it, such as by changing attitudes and opinions.

Undoubtedly, the co-occurrence of trust in science with conspiracy thinking can produce a sense of psychological contradiction of one’s beliefs. Levandowsky, Gignac, and Oberauer [7] highlight the multiple cognitive reasons why conspiracy ideology is in opposition to the scientific method. This contradiction does not necessarily lead to the modification of one’s beliefs, especially if one’s self-image is not threatened. We assume that this co-occurrence of trust in science with conspiracy thinking creates uncertainty and indecision both at the stage of expressing vaccination intentions and at the stage of taking action.

### 4.3. Differences between Groups in the Level of Individual Variables

The declared intention to vaccinate against COVID-19 was associated with a higher trust in science and a lower level of conspiratorial thinking. Religiosity was slightly lower in the willing-to-vaccinate group than the undecided group, but no clear conclusions can be drawn. This is consistent with existing theories indicating that anti-scientific beliefs and religiosity are related to general vaccine hesitancy and rejection, and COVID-19 vaccines are no different in this regard.

The same effects are true for actual vaccination against COVID-19, as the vaccinated group exhibited the highest trust in science and the lowest level of conspiratorial thinking, the unvaccinated group had the lowest trust in science and highest level of conspiratorial thinking, and the undecided were in the middle.

The consistency between intention and behavior followed a similar pattern—consistent acceptance of the COVID-19 vaccine (intended and actual uptake) was associated with lower conspiratorial thinking and higher trust in science, as well as a slightly lower religiousness than in the undecided group.

This pattern of results strongly indicates that trust in science and conspiratorial thinking are crucial factors influencing the willingness to vaccinate, both declared and actual, while religiousness does not seem to be an equally useful predictor, which is consistent with existing research that religious orthodoxy, rather than general religiousness, is a predictor of vaccine rejection.

The change in conspiratorial thinking and trust in science over time also shows an interesting pattern. We observed a decrease in conspiratorial thinking in consistent vaccine rejecters, while there was an increase in trust in science in consistent vaccine supporters. Moreover, those who turned from undecided to not vaccinated also exhibited a decrease in trust in science.

## 5. Conclusions

The lowest trust in science and the highest level of conspiracy thinking was observed in those who rejected vaccination. In the entire sample in the first and second measurements, there was a positive correlation between religiosity, general conspiracy thinking and low trust in science. One important factor in being undecided at the declaration stage and at the vaccination stage may be the presence of a positive relationship between trust in science and conspiracy thinking in some respondents. The positive effect of trust in science on pro-vaccine attitudes was confirmed when we tested whether there was a change in attitudes toward science and conspiracy thinking between the first and second measurements—there was an increase in trust in science among consistent proponents of vaccination, and a decrease in trust in science among those who switched from undecided to unvaccinated. In sum, trust in science may be considered a factor leading to COVID vaccine acceptance, while conspiratorial thinking may lead to COVID vaccine rejection. Also, results indicate that measuring the declared willingness to vaccinate may not reflect actual vaccination behavior, especially when considering anti-vaccine or undecided individuals.

The obtained results indicate the need to investigate other individual variables which may lead to COVID vaccine rejection and especially the inconsistency between intent and action, as possibilities may exist to influence the anti-vaccine and undecided population to vaccinate. While the current study concerns COVID-19 vaccination specifically, existing theory allows for the expectation that these results may apply to other vaccines as well; however, a direct empirical confirmation is needed.

## Figures and Tables

**Table 1:** Means and standard deviations of individual differences between groups.

Group (Declaration /Action)	Conspiratorial Thinking	Trust in Science	Religiosity
NO/NO	M = 3.75, SD = 0.72	M = 3.43, SD = 0.91	M = 2.77, SD = 1.19
NO/UND	M = 3.37, SD = 0.79	M = 3.58, SD = 0.81	M = 2.80, SD = 0.97
NO/YES	M = 3.73, SD = 0.82	M = 3.58, SD = 0.57	M = 2.60, SD = 1.06
UND/NO	M = 3.65, SD = 0.65	M = 3.53, SD = 0.80	M = 2.88, SD = 0.98
UND/UND	M = 3.55, SD = 0.59	M = 3.54, SD = 0.60	M = 3.02, SD = 1.11
UND/YES	M = 3.40, SD = 0.71	M = 3.66, SD = 0.69	M = 2.98, SD = 0.99
YES/YES	M = 2.96, SD = 0.87	M = 4.03, SD = 0.67	M = 2.50, SD = 1.22

## Data Availability

Data is available in OSF repository: https://osf.io/q3yh4/?view_only=cdd9e7b6d13744e3911ac0883d843066 (accessed on 22 December 2022).
